# Engineering molecularly imprinted polymers for receptor-specific cancer therapeutics

**DOI:** 10.1039/d6nr00511j

**Published:** 2026-04-14

**Authors:** Shreya Tiwari, Charles L. Hutchinson, Pankaj Singla, Robert C. Rintoul, Timothy H. Witney, Nicholas W. Turner, Marloes Peeters

**Affiliations:** a Department of Chemical Engineering, University of Manchester Nancy Rothwell Building Manchester M13 9QS UK marloes.peeters@manchester.ac.uk; b Department of Oncology, University of Cambridge Cambridge CB2 0QQ UK; c School of Biomedical Engineering & Imaging Sciences, King's College London London SE1 7EH UK; d Chemistry, School of Mathematical and Physical Sciences, University of Sheffield Sheffield S3 7HF UK

## Abstract

The therapeutic efficacy of cancer therapeutics is frequently limited by poor tumour selectivity, systemic toxicity, and the emergence of drug resistance, underscoring the need for advanced nanoscale drug delivery systems (DDSs) capable of precise molecular targeting and controlled release. Molecularly imprinted polymers (MIPs) have emerged as a promising class of synthetic nanocarriers that combine highly selective and programmable molecular recognition with the robustness, tunability, and scalability of polymeric materials. This review examines recent advances in the nanoscale engineering of MIPs for receptor-guided precision cancer therapy, focusing on how imprinting strategy, polymerisation methods, and nanostructure control govern binding affinity, selectivity, and drug-release behaviour. Key advances in epitope imprinting are highlighted to overcome the size, conformational complexity, and stability challenges associated with whole-protein templates, enabling reproducible targeting of cancer-associated receptors. Emerging stimuli-responsive, hybrid, and multifunctional MIP architectures are discussed, illustrating how molecular recognition, drug loading, and triggered release can be co-engineered within a single nanoscale platform. Finally, the current challenges related to biocompatibility, reproducibility, and translation towards manufacturable and regulatory-compliant systems are critically assessed, outlining future directions for establishing MIPs as a viable class of next-generation precision DDSs in oncology.

## Abstract 2

Cancer therapy is often limited by poor tumour selectivity, systemic toxicity, and drug resistance, highlighting the need for advanced nanoscale drug delivery systems that enable precise molecular targeting and controlled therapeutic release.

## Graphical abstract

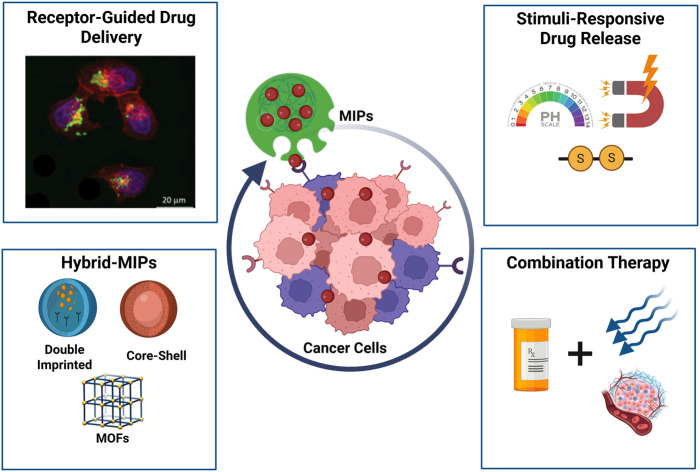

## 1. Introduction to drug delivery

Cancer is characterised by the uncontrolled growth and spread of abnormal cells in the body. According to Cancer Research UK (CRUK), an estimated 18.1 million new cancer cases were diagnosed worldwide in 2020, with approximately 10 million cancer-related deaths reported during the same year.^1^ Over recent decades, substantial advances in cancer treatment have been achieved, partly due to a better understanding of cell biology and the tumour microenvironment. Current treatment modalities include chemotherapy, immunotherapy, radiotherapy, and surgery, with chemotherapy and chemoimmunotherapy being the main systemic approaches. Despite these advances, major challenges persist, including drug resistance, limited tumour specificity resulting in off-target effects, and treatment-related cytotoxicity. Moreover, patients who initially respond often relapse with recurrent disease.^2^ In such cases, the treatment might then proceed with trying a different regimen of drugs or a combination of drugs, which would further increase the harmful effects but not significantly improve the patient's condition.^3^ The fundamental mechanism of many chemotherapeutic agents is to target rapidly dividing cells, rendering them inherently toxic to healthy proliferating tissues such as bone marrow, gastrointestinal epithelium, and hair follicles.^4^ Consequently, treatment failure is frequently associated not only with insufficient drug potency, but also with suboptimal drug delivery, poor pharmacokinetics, and lack of tumour-selective accumulation.^5–7^

As an alternative, antibody-based therapies offer improved selectivity as both stand-alone therapeutics, co-factors or carriers. Antibodies are immunoglobulin proteins engineered to recognise tumour-associated antigens and, as such, can selectively target cancer cells for immune-mediated destruction. While antibodies offer high affinity, selectivity, and broad clinical applications, they do have some limitations, including immunogenicity, limited stability, susceptibility to enzymatic degradation, and complex, high-cost manufacturing and purification processes.^8^ To address these drawbacks, a range of non-antibody platforms has been explored to perform the same functionality. These include nanobodies (engineered antibody fragments),^9^ affibodies,^10^ and DARPins (synthetic proteins), as well as non-antibody scaffold drugs,^11^ and aptamers (single-stranded nucleic acids).^11^ These alternatives often offer better solubility, tissue penetration, stability, and comparatively lower production costs than antibodies; however, each class is still associated with its own set of challenges.^12,13^

The effectiveness of cancer treatment can be significantly improved by site-specific drug release. To this end, drug delivery systems (DDSs) have been developed to enhance the treatment efficiency and mitigate the harmful side effects of chemotherapy. DDSs work by delivering high concentrations of chemotherapeutic drugs to the tumours while limiting exposure to healthy tissues, thereby reducing adverse effects and improving patient outcomes.^14^ These systems commonly employ nanoscale carriers capable of encapsulating or conjugating therapeutic agents and enabling controlled, localised release. Nanocarriers offer several advantages over conventional chemotherapy, including improved drug solubility, enhanced stability, prolonged circulation time, and preferential tumour accumulation through the enhanced permeability and retention (EPR) effect. Following tumour accumulation and cellular uptake, efficient endosomal escape prevents intracellular degradation and enables therapeutics to reach their intended intracellular targets, thereby enhancing treatment efficacy.^15^

A wide range of nanomaterials has been investigated for DDSs’ design, including polymers,^16,17^ lipids,^18,19^ and inorganic materials.^20,21^ Several nanomedicines have achieved clinical translation, including Doxil® (liposomal doxorubicin), Abraxane® (albumin-bound paclitaxel), Lupron Depot® (PLGA microspheres for leuprolide), Oncaspar® (PEG-asparaginase), Zoladex® (biodegradable goserelin implant), DepoDur™ (extended-release morphine in a polymer-based system), and Eligard® (leuprolide acetate in ATRIGEL® polymer matrix)^22^ as discussed in Table 1. In parallel, nanobiomaterials have emerged as an important class of nanocarriers engineered to interact with biological systems. Their unique physicochemical properties, high surface area, and tunable surface chemistry enable efficient loading of therapeutic agents and controlled interactions with cells and tissues, making them versatile platforms for drug delivery, diagnostic and targeted therapies.^23,24^

**Table 1:** DDSs in cancer therapy: types, mechanisms and key advantages, endosomal escape strategies, preclinical and clinical evidence, targeting levels, and key attributes

DDS type	Mechanism/key advantages	Endosomal escape (EE) role	Preclinical evidence	Clinical/commercial examples	Targeting level	Main characteristics
Liposomes (PEGylated; pH-/thermo-sensitive)	Encapsulate hydrophilic/hydrophobic drugs; improve solubility and PK *via* PEGylation; stimuli-responsive release (pH, hyperthermia)	Critical for intracellular delivery; pH- and thermo-sensitive designs enhance cytosolic release	Functionalised liposomes, including immunoliposomes and pH-/thermosensitive types, improve DOX delivery^79^	Doxil®, Caelyx®, Myocet®, Onivyde®, Vyxeos®; LTLD in trials^80^	Passive (EPR) ± active (ligand-decorated)	Selectivity: medium to high; solubility: high; stability: medium to high
Albumin-bound nanoparticles	Solvent-free delivery of hydrophobic drugs *via* albumin pathway; altered biodistribution	EE not primary; uptake *via* caveolae transcytosis; some endosomal trafficking occurs	Improved PK/efficacy *vs.* Cremophor-paclitaxel; caveolae-mediated transport implicated^81^	Abraxane® (nab-paclitaxel)^82^	Passive (EPR) + albumin pathway	Selectivity: medium; solubility: high; stability: high
Polymeric nanoparticles/microparticles (PLGA, ATRIGEL®, micelles)	Controlled release; tunable degradation; high loading; improved PK/PD	Important when intracellular action required; proton-sponge/pH-responsive polymers can promote EE^83^	PLGA microspheres sustain leuprolide; PEG-PLA micelles (Genexol-PM) enhance paclitaxel delivery and efficacy^83^	Lupron Depot®, Eligard®, Genexol-PM^84^	Passive (EPR) ± active	Selectivity: medium; solubility: high; stability: high
PEGylated proteins/polymer conjugates	Increase half-life; reduce immunogenicity; improve PK	EE not central (systemic/extracellular enzymatic action)	PEG-asparaginase demonstrates improved PK and reduced immunogenicity *vs.* native enzyme^85^	Oncaspar®^86^	Systemic (non-targeted)	Selectivity: low; solubility: N/A; stability: high
Biodegradable implants	Localised, sustained release; improved compliance; reduced systemic toxicity	EE not applicable (local delivery)	Goserelin implants provide controlled hormonal suppression *in vivo*^87^	Zoladex®^88^	Local delivery	Selectivity: high; solubility: N/A; stability: high
Lipid nanoparticles (LNPs) for RNA	Ionizable lipids protect nucleic acids; optimised for EE *via* pH-triggered membrane interactions	EE is major barrier (<5% escape without design); ionizable/cationic lipids and EV-inspired strategies improve EE.	Structure–function evolution in ionizable lipids improves mRNA cytosolic release^89^	Multiple cancer mRNA nanovaccines in trials; no FDA-approved cancer LNP yet	Passive (EPR) ± Active (ligands)	Selectivity: medium to high; solubility: N/A; stability: medium to high
Inorganic nanoparticles (radio/thermotherapy)	Hafnium oxide (radioenhancer); iron oxide (magnetic hyperthermia)	EE not relevant (extracellular/physical modality).	Validated clinical use; tumour ablation/RT enhancement^90^	Hensify® (NBTXR3), NanoTherm®^91,92^	Physical targeting (field-driven)	Selectivity: medium; solubility: N/A; stability: high
Molecularly Imprinted Polymers (MIPs)/nanoMIPs	Synthetic epitope/receptor recognition; high stability; cost-effective; dual-template imprinting co-loads drug	Required for intracellular targets; pH-responsive monomers aid EE.	nanoMIPs targeting EGFR and ERα enable selective binding and functional inhibition; bispecific VEGF/DLL4 suppress MCF-7 tumours; dual-imprint EGFR + DOX systems achieve targeted delivery^16,93^	Preclinical stage only^28^	Active (epitope/receptor)	Selectivity: high; solubility: formulation-dependent; stability: high

Among the nanocarrier platforms, polymeric nanoparticles are of particular interest due to their structural versatility and tunable physicochemical properties. Their high surface-to-volume ratio enables efficient drug loading and functionalisation with targeting ligands, while their degradability and mechanical flexibility allow for controlled release and reduced systemic toxicity.^25^

One emerging class of polymeric nanocarriers with significant promise for cancer drug delivery is molecularly imprinted polymers (MIPs), particularly nanoMIPs (single-particle MIPs with diameters of sub-250 nm). MIPs are synthetic materials engineered with specific binding sites that are complementary in shape, size, and functional groups to a target molecule, enabling selective recognition and rebinding. MIPs are fully capable of selective molecular recognition, offering a robust alternative to biological targeting ligands for drug delivery applications.^26^ Their high chemical stability, resistance to enzymatic degradation, and long shelf life make them particularly attractive for use in physiologically demanding environments.^27^ Importantly, advances over the past ten years have enabled MIPs to be engineered into a nanoplatform, nanoMIPs. nanoMIPs can be generated to recognise cancer-relevant targets, including overexpressed cell-surface receptors and biomarkers, peptide epitopes, and tumour-associated microenvironmental cues, enabling preferential tumour localisation and controlled drug release.^16,28^ These attributes position MIPs as a versatile and adaptable platform for developing DDSs.

This review focuses on the development of MIPs-based DDSs for cancer therapy, emphasising cancer-specific recognition and controlled therapeutic administration. It critically evaluates imprinting strategies, rational carrier design, and receptor-guided approaches for precise drug delivery. In addition, key advances in MIPs-based delivery architectures, including hybrid systems and combinational therapeutic platforms, are discussed alongside biocompatibility considerations, scale-up challenges, and translational barriers. Overall, this work aims to highlight the potential of MIPs as next-generation nanocarriers for selective, safe, and effective DDSs.

## 2. Design and engineering of MIPs for drug delivery

MIPs are best described as synthetic molecular recognition materials that mimic the selective binding properties of natural antibodies, achieved through templated polymerisation. This interaction is best envisaged by the classical ‘lock and key’ mechanism, where the polymer selectively binds with the molecule that was imprinted within it during its formation,^29^ though naturally this is an analogy, and the reality of recognition is far more complex, with an induced fit system commonly theorised. They have found applications in a range of fields, including (bio)sensing, separations and catalysis; however, since the inception of nanoMIPs design, they have emerged as selective drug-carriers capable of providing targeting, high affinity/selectivity, stability, and controlled release through their tailor-made binding cavities.^28,30^ Their robustness, low immunogenicity, and tunable physicochemical properties can enable the protection of loaded therapeutics while allowing for targeted delivery to tissues with imprinted biomarkers or microenvironment conditions.^28,31,32^ Recent advances in MIPs-based DDSs design, including structural modifications, epitope imprinting, stimuli responsiveness, and combinational therapies, have further enhanced their potential in cancer therapy.

A general synthesis method for MIPs involves forming a pre-polymerisation complex with the template and complementary monomers. Subsequently, the polymerisation is carried out in the presence of an initiator and a crosslinker. Finally, the template molecule is extracted from the polymer, leaving behind complementary cavities that are specific to the template molecule, resulting in the production of a fixed, target-specific binding site entrapped with a polymer matrix.^33^ The nanoMIPs offer the advantages of nanomaterials and can rival the affinity of natural receptors, in addition to offering good biocompatibility.^16,34^

### 2.1. Imprinting methods and polymerisation techniques

Based on the molecular interactions involved in the formation of the pre-polymerisation complex, the synthesis of MIPs can be categorised as covalent, non-covalent or semi-covalent. In the covalent approach, reversible covalent bonds form between the template and monomers during polymerisation and are later cleaved to remove the template, generating homogeneous binding sites with reduced non-specific interactions. However, this approach is limited by the difficulty of identifying suitable reversible template-monomer systems and by slower binding kinetics associated with covalent interactions, which can hinder equilibrium binding.^35,36^ When designing a DDS, the key limitation of covalent imprinting is that only a few drugs with suitable functional groups can be used, and covalent binding may reduce their therapeutic activity.^37^

The non-covalent approach, also known as the self-assembly method, involves non-covalent interactions between the template and monomers prior to polymerisation, including hydrogen bonds, hydrophobic interactions, ionic, electrostatic, and van der Waals forces. This method is far more prevalent (>95% of examples) due to its simplicity, flexibility and ease of template removal. Additionally, in terms of rebinding kinetics, the non-covalent approach often gives better results. However, imprinting efficiency can be sensitive to disruptions in template-monomer interactions.^36,38^ For example, a comparison for stigmasterol (a plant sterol with anti-inflammatory, neuroprotective, and anti-tumour properties) showed that non-covalent imprinting is synthetically simpler but yields lower-affinity MIPs due to unstable hydrogen bonds, whereas covalent imprinting provides stronger binding, less cross-reactivity, and higher imprinting efficiency.^39^ Despite this, most MIPs-based DDSs favour the non-covalent approach using acrylic monomers, often with minimal optimisation, as it allows versatile and successful imprinting of a wide range of chemotherapeutic drugs.^37^ The semi-covalent approach combines covalent template attachment during polymerisation with non-covalent rebinding, but its use remains limited due to increased synthetic complexity.^40,41^

One of the primary advantages of MIPs is their versatility in forming different material architectures, which has led to the development of multiple synthetic strategies. In bulk imprinting, a mixture of template, monomers, and cross-linkers is polymerised to form a monolithic polymer, which is subsequently ground into particles. Although straightforward, this method often produces irregular, polydisperse particles with partially inaccessible binding sites, reducing binding capacity and selectivity.^42,43^ Surface imprinting generates recognition sites at or near the surface of a support material, improving site accessibility, mass transfer and binding kinetics, and is particularly suitable for biomolecule imprinting.^42,44,45^ However, it is limited to applications beyond sensing and separations. These are but two strategies in a myriad of methods that have been explored.

Solid-phase supported imprinting has recently emerged as an alternative strategy in which the template is immobilised on a solid support during polymerisation. This approach facilitates the synthesis of uniform nanoMIPs with improved binding-site homogeneity, reduced template leaching and enhanced reproducibility, making it particularly suited for drug-delivery applications.^46^

Among the various polymerisation reactions used for synthesising MIPs, free radical polymerisation (FRP) remains the most widely employed. FRP involves radical-initiated chain-growth polymerisation of unsaturated monomers and is widely used due to its simplicity, scalability, compatibility with a broad range of monomers and solvents, and ability to proceed under mild conditions, including aqueous environments suitable for biological targets.^47–49^ Consequently, FRP is prevalent in the synthesis of solid-phase nanoMIPs.^46^ However, FRP provides limited control over polymer growth, which can lead to heterogeneous binding sites and branched polymer structures that reduce affinity and selectivity.^41,50^ Controlled radical polymerisation (CRP) techniques have therefore been developed to improve control over polymer architecture and molecular weight, often resulting in more homogeneous networks and more predictable drug-release behaviour.^51^ However, FRP remains the dominant approach due to its simpler procedure, broad applicability to a wide range of monomers and templates, and ease of implementation without specialised catalysts or chain-transfer agents.^52^

### 2.2. Rational design of MIPs

During the pre-polymerisation stage, functional monomers associate with the template through complementary non-covalent interactions, forming a template-monomer complex, with monomer functionality, stoichiometry, and spatial arrangement influencing the formation of recognition sites. Subsequently, polymerisation entraps these complexes within a crosslinked polymer network, providing mechanical stability and maintaining the integrity of the imprinted recognition cavities. As a result, characterising the orientation and the strength of monomer-template interactions in the pre-polymerisation has become a central strategy for informing rational MIPs design.^53^ However, the polymerisation process and cross-linking inevitably alter local spatial arrangements, polarity, and accessibility of binding sites, meaning that pre-polymerisation models capture important information but do not fully reproduce the complexity of the final MIPs binding environment.^41,54^

The pre-polymerisation complex can be probed experimentally using techniques such as UV-Vis or fluorescence spectroscopy, as well as Fourier-transform infrared (FTIR) spectroscopy and Nuclear Magnetic Resonance (NMR), which provide qualitative and semi-quantitative insight into monomer-template interactions.^55,56^ Post-polymerisation techniques, including isothermal titration calorimetry (ITC) and surface plasmon resonance (SPR), allow quantitative evaluation of binding affinities and kinetics through association and dissociation constants.^57,58^ While these methods provide valuable experimental validation, systematic screening of monomer-template combinations can be time-consuming and resource-intensive.^33^

Computational methods, therefore, play a complementary role to experimental screening by providing a rapid, cost-effective means to predict and rank monomer-template interactions prior to synthesis.^59^ Modelling large and heterogeneous systems, including polymers and proteins, remains computationally challenging.^60^ Consequently, computational approaches typically focus on simplified representations, such as epitopes rather than full proteins, and pre-polymerisation complexes rather than fully formed MIPs structures.^61^

Molecular docking is widely used to predict favourable binding geometries, identify binding regions on the template, and rapidly screen large libraries of functional monomers. This approach has been successfully applied to small-molecule drugs, guiding the selection of functional monomers for high-affinity binding, as well as to biomolecular targets such as bovine serum albumin (BSA) epitopes, where docking helps identify key amino acid residues for targeted monomer interactions and rational polymer design.^62,63^ Molecular dynamics (MD) simulations extend this analysis by incorporating solvent effects, temperature and molecular motion, providing insight into the stability and persistence of binding interactions.^64^ MD studies in MIPs design have highlighted the critical influence of solvent choice on complex stability and binding-site formation.^65^ By capturing conformational flexibility and dynamic rearrangements, MD offers an important refinement step beyond static docking predictions. Density Functional Theory (DFT) provides a complementary quantum-mechanical description of specific interactions, enabling accurate calculation of binding energies within pre-polymerisation complexes and facilitating quantitative comparison of functional monomers, crosslinker monomers, and solvents.^66,67^ For example, DFT analyses have been used to identify acrylic acid as an optimal functional monomer, ethylene glycol dimethacrylate as an effective crosslinker monomer, and toluene as a suitable porogenic solvent for developing a high-specificity imprinted polymer, targeting the small-molecule drug oxybutynin.^68^ Despite its accuracy, DFT is not readily applicable to large-scale MIPs screening because of its unfavourable computational scaling with system size.^61^ Together, docking, MD and DFT form a complementary computational framework for rational MIP design.

Experimental and computational approaches, therefore, provide complementary advantages.^61^ Analytical methods provide definitive insight into material performance by using empirically determined parameters that reflect the system's true behaviour, but they are typically labour-intensive and costly.^56,69^ Computational modelling, whilst unable to provide definitive experimental validation, enables the high-throughput exploration of theoretical design space, composition and synthesis conditions.^70^ Recent studies show that integrating *in silico* screening with rapid experimental validation can establish more efficient design-build-test cycles for MIPs development.^71^ For example, multi-monomer docking strategies combined with high-throughput fluorescence binding assays have been used to prioritise monomer compositions for MIPs targeting SARS-CoV-2, showing strong agreement between computational predictions and experimentally measured binding performance.^72^ Addressing this central knowledge gap, the lack of a scalable, predictive framework for rational MIPs design, therefore, requires hybrid, multi-fidelity approaches that combine quantum accuracy with efficient exploration of high-dimensional design spaces.^73^ This challenge is particularly acute in the design of MIPs for drug delivery, where trade-offs must be balanced between optimising the affinity of the materials for specific receptors, enabling efficient drug incorporation into the polymer matrix, and ensuring the biocompatibility of the final material.

Additional considerations further complicate the rational design of MIPs for drug delivery. Template stability is a key factor, as peptides and biomolecules are prone to degradation under some polymerisation conditions (*e.g.* elevated temperatures, buffered solutions, ionic environments).^74^ Moreover, many drug-like templates and inhibitors exhibit inherent instability or toxicity, thereby limiting their suitability for *in vivo* applications. Crosslinkers influence affinity not only by stabilising the polymer network but also by introducing additional binding-site interactions and modulating the physicochemical properties of the pre-polymerisation complex, such as hydrophobicity. The choice and amount of crosslinkers, therefore, represent a trade-off between polymer stability and the flexibility needed to optimise binding site formation.^75,76^ For example, in designing DDSs, lower degrees of crosslinking are preferred, as it allows for more tunable and controlled drug release, with drug loading capacity emerging as a key parameter.^26^ Porogenic solvents further dictate MIP morphology and pore structure, while also influencing the stability of non-covalent template-monomer interactions during polymerisations.^56,77^

### 2.3. Comparative advantages of MIPs in designing DDSs

MIPs possess a unique combination of physicochemical and molecular recognition properties, making them attractive candidates for biomedical applications, particularly in the development of DDSs. Their high drug loading capacity, structural and chemical stability, tunable crosslinking density, and strong affinity toward target molecules enable efficient encapsulation, retention, and controlled release of drugs. Collectively, these characteristics facilitate improved targeting of diseased sites and enhance the potential of MIPs as advanced carriers for therapeutic delivery.^26,37,78^ As summarised in Table 1 MIPs uniquely combine receptor-level molecular recognition with the robustness and tunability of synthetic polymers, distinguishing them from conventional drug delivery platforms.

One of the most significant advantages of MIPs is their selectivity towards target molecules, arising from the formation of complementary cavities during polymerisation, enabling MIPs to function as synthetic receptors with antibody-like selectivity and enhanced robustness.^94,95^ This molecular specificity fundamentally distinguishes MIPs from conventional polymeric nanocarriers, which typically rely on non-specific interactions for drug loading and targeting. Additionally, MIPs exhibit versatility in targeting different templates, uniquely enabling recognition of both small molecules and macromolecules, capabilities that antibodies often cannot achieve.^31,96^

By imprinting peptides, proteins, or receptor-specific ligands, MIPs can be engineered to selectively bind overexpressed receptors on cancer cells, facilitating receptor-mediated targeting and enhanced cellular uptake. For instance, nanoMIPs imprinted against a breast cancer marker have demonstrated antibody-comparable affinity while facilitating the endocytosis and intracellular delivery of doxorubicin (DOX) in ERα-overexpressing cancer cells.^16^ In this way, MIPs can serve both to recognise target cells and to retain the loaded drug within the polymer matrix, making them particularly beneficial for the design of efficient DDSs. Additionally, this affinity-driven recognition enables preferential binding and stable retention of therapeutic agents within the polymer matrix, even in complex biological environments. This selectivity enhances drug loading efficiency, reduces premature drug leakage, and promotes targeted drug accumulation, thereby improving therapeutic efficacy and reducing systemic toxicity.^97^ Moreover, MIPs can be engineered to remain structurally stable under physiological conditions (pH 7.4) while exhibiting pH-responsive behaviour in the acidic tumour microenvironment (pH 6.5), which minimises premature drug loss during systemic circulation.^98^

A critical comparison with established targeting ligands further contextualises the roles of MIPs in the design of DDSs. While monoclonal antibodies (mAbs) and antibody–drug conjugates (ADCs) are widely used for active targeting due to their high affinity and specificity, nanoMIPs have been shown to have *K*_D_ values in the nanomolar range. For example, a comparative study using SPR and electrochemical impedance spectroscopy (EIS) showed that nanoMIPs synthesised against the protein antigen bovine haemoglobin reported binding affinities of 38 pM (EIS) and 3.1 pM (SPR), while the corresponding polyclonal antibody exhibited affinities of 52 pM (EIS) and 49 nM (SPR), demonstrating that nanoMIPs can rival antibody-based recognition in specific systems.^99^ Other studies have shown that MIPs enable precise detection of target analytes at picomolar concentrations.^100^ Moreover, mAbs are large protein molecules (∼150 kDa) protein molecules that exhibit limited tissue penetration^101^ and are susceptible to denaturation or enzymatic degradation, whereas MIPs are synthetic polymeric materials with greater physicochemical stability and tunable structural properties. However, unlike antibodies, whose pharmacokinetics and biodistribution are well characterised, the *in vivo* fate and long-term clearance mechanisms of MIPs remain less characterised. In this context, MIPs represent a complementary class of synthetic recognition elements that combine receptor-level molecular recognition with the robustness and design flexibility of synthetic polymers.

Unlike their biological counterparts, MIPs are stable over a broad range of pH, temperature and chemical environments, allowing them to maintain structural integrity during circulation and exposure to complex biological media. This stability enables MIPs to protect encapsulated drugs from premature degradation following administration, thereby preserving therapeutic activity until delivery to the target site.^102,103^ Robustness is critical for DDSs that require site-specific action and precise dosing, while also enabling the integration of stimuli-responsive features for controlled drug release in response to physiological triggers.^102,103^ These properties of MIPs also support formulation stability,^37,104,105^ a crucial consideration for clinical translations of these systems. However, the rational design of biodegradable MIPs offers a promising strategy to minimise systemic accumulation and facilitate safe clearance, addressing growing concerns over the long-term persistence of non-degradable nanocarriers.^106^ Furthermore, MIPs adhere to the 3Rs by *replacing* animal-derived antibodies, *reducing* the use of animals for immunisation, and *refining* experimental design to minimise *in vivo* interventions.^107^

The versatility of MIPs further extends to their synthesis and design. As briefly discussed above, they can be prepared in various morphologies such as nanoparticles, thin films, and bulk hydrogels, enabling their properties to be tailored to specific applications. Key parameters such as particle size, crosslinking density, and surface chemistry can be systematically tuned to optimise drug loading, release behaviour, and biological interactions.^108^ Multiple well-established synthesis protocols exist, making their production relatively straightforward and reproducible.^33,109^ This reproducibility supports batch-to-batch consistency, which is critical for pharmaceutical development and scale-up. Additionally, MIPs can be readily integrated with other functional components, enabling the development of multifunctional drug delivery platforms,^106,110,111^ as discussed later in this review.

Cost-effectiveness represents another important advantage of MIPs. They are typically synthesised from inexpensive monomers and crosslinkers using simple polymerisation methods that do not require sophisticated equipment or complex production conditions.^112,113^ For example, MIPs synthesised *via* solid-phase synthesis for multiple templates exhibited picomolar-level affinity, acceptable cross-reactivity, and retained binding performance after storage at room temperature for extended periods.^100^ These studies also highlight the mechanical and chemical robustness of MIPs, which contributes to their long shelf life. MIPs can be stored in a dried state without loss of functionality and can withstand harsh conditions, with analytical characterisation confirming preservation of specificity and structural integrity over several months. Additionally, preservation approaches such as lyophilisation and autoclaving do not affect performance.^100,114,115^ For drug delivery applications, MIPs are commonly synthesised using acrylate or (meth)acrylate-based functional monomers and crosslinkers with favourable biocompatibility profiles; however, comprehensive evaluation of their long-term toxicity and *in vivo* fate remains an important area for future investigation.^73,108^

## 3. Cancer receptors-guided targeted therapy

### 3.1. Targeting cancer receptors

Tumours possessed distinct molecular and anatomical features that differentiate them from normal, healthy tissue. Anatomical features of tumour biology include increased blood vessel leakiness and branching, enabling systemic drug delivery to cancer cells *via* the EPR effect, a passive mechanism for drug delivery.^116,117^ Cancer cells often overexpress specific receptors or antigens on their surface, which support their proliferation and survival.^118^ Active targeting is a strategy often employed within DDSs, which is achieved by specific recognition of targeted ligands to cancer-specific cell surface receptors, followed by internalisation *via* receptor-mediated endocytosis and subsequent release of the therapeutic to the cell.^119,120^ The advantages of this approach include targeted drug delivery to the tumour site, reducing toxicity *via* minimising exposure to healthy cells, enhanced efficacy, and the potential to bypass certain multidrug resistance mechanisms.^121,122^ mAbs^123^ and ADCs,^124^ which have revolutionised cancer care and are a standard part of treatment for several types of cancer, primarily work through this mechanism. Despite several ADCs demonstrating clinical utility, approximately 90% of ADC candidates and compounds fail in late-stage clinical trials, suggesting that alternatives are needed.^125^ The clinical implementation of MIP strategies, however, remains underexplored due to tumour heterogeneity, complexity in the tumour microenvironment, and difficulties in implementing large-scale strategies that facilitate the precise manufacturing of the developed nanocarriers.^126–129^

#### 3.1.1. Roles and types of receptors in targeted drug delivery

Overexpressed cancer receptors range from growth factor receptors and nutrient transporters (such as folate and transferrin receptors) to hormones, cell adhesion molecules, cytokines, immune checkpoints, and developmental markers such as Claudins, depending on the type of cancer.^130,131^ MIPs have been designed to selectively target these receptors for cancer therapy, as summarised in Table 2. A rapidly expanding area is the use of immune checkpoints to guide therapeutic payloads into the tumour microenvironment (TME), aiming to overcome resistance to checkpoint blockade inhibitors. Immune checkpoints are inhibitory receptors and ligands that regulate T cell activation, maintain immune homeostasis, and prevent autoimmunity. Common immune checkpoints include programmed death ligand 1 (PD-L1), programmed cell death protein 1 (PD-1),^132^ cytotoxic T-lymphocyte-associated protein 4 (CTLA-4), and lymphocyte activation gene 3 (LAG-3).^130^

**Table 2:** Overview of molecularly imprinted polymers developed for targeting cancer-associated biomolecules, including receptors, carbohydrates, growth factors, vitamins, cytokines, and immune checkpoints, with their functional roles

Receptor family	Receptor type	Example	Functional role/targeting advantage	MIP applications
Vitamin transporter	Folate receptor	FRα	Highly overexpressed in breast, ovarian, kidney, lung, and brain cancers; minimal in normal cells	MIPs targeting FRα loaded with DOX or methylene blue for HeLa xenografts;^138^ paclitaxel-loaded folate-functionalized MIPs for MDA-MB-231 cells (IC_50_ 4.86 nM *vs.* 32.80 nM)^137^
Lectin family	Lectin	Galectins	Promote tumour growth and immune evasion	Hairy fluorescent MIPs imprinted with SA for 5-FU delivery and imaging^143^
		C-type lectins	Requires Ca^2+^ for carbohydrate binding; overexpressed on tumour-associated immune cells	Carbon dot-core MIPs targeting glucuronic acid for cervical cancer imaging^144^
		Selectins	Mediate adhesion and metastasis	MIPs for selectin-targeted delivery (conceptual)
Carbohydrate target	Monosaccharide	Sialic acid	Enable receptor-mediated uptake *via* lectin overexpression; improve solubility and tumour penetration	Multi-responsive MIPs for SA (5-FU delivery)^143^
		Glucuronic acid	Target hyaluronan substructure overexpressed on cancer cells	Carbon dot-core MIPs for glucuronic acid imaging^144^
Growth factor receptor family	Growth factor receptor	VEGF	Overexpressed in aggressive cancers such as glioblastomas; mediates endocytosis	VEGF-imprinted MIPs with quantum dots for zebrafish xenografts;^99^ bispecific MIPs for VEGF & DLL4 (68–72% tumour suppression)^146^
		EGFR	Associated with aggressive phenotypes; receptor-mediated uptake	EGFR-imprinted nanoMIPs with DOX (*K*_D_ 3.6 nM);^101^ dopamine-based EGFR-imprinted MIPs for pH-responsive DOX release^147^
		HER2	Overexpressed in breast and ovarian cancers; mediates endocytosis	HER2-targeted MIPs for DOX delivery and tumour suppression^103^
		ERα	Hormone receptor; nuclear delivery *via* receptor-driven uptake	ERα-targeted MIPs with DOX and fluorescent dye (*K*_D_ 14.7 nM)^16^
Vitamin	Biotin	Biotin	Essential for rapidly dividing cancer cells; promotes growth	Biotin-functionalized core–shell MIPs for MCF-7 cells (DOX delivery, viability reduced from 90% to 60%);^149^ biotin-selective MIPs binding biotinylated CD44 aptamer^150^
Cytokine	Interleukin	IL-6	Stimulate growth and invasiveness in multiple cancers	Molecularly imprinted nanotraps for IL-6 sequestration, reducing extracellular IL-6 levels^152^
Immune checkpoint	Immune checkpoint	PD-L1	Guide therapeutic payloads into TME; overcome resistance to checkpoint inhibitors	Emerging concept: MIPs targeting PD-L1 for enhanced immunotherapy^132^
		PD-1	Immune regulation: target for immunotherapy	Emerging concept: MIPs targeting PD-1^132^

In addition to immune checkpoints, several receptors are frequently overexpressed in a wide range of cancer types, including folate receptors (FRs), lectins, monosaccharides, growth factor receptors (*e.g.*, vascular endothelial growth factor (VEGF), epidermal growth factor receptor (EGFR), estrogen receptor (ER), and human epithelial growth factor receptor (HER2)), biotin, and interleukins.^131,133–135^ FRs are overexpressed in various cancers, including breast, ovarian, kidney, lung, brain, and epithelial tumours, while being largely absent from normal cell membranes.^136^ MIPs have been engineered to recognise conformationally flexible regions of FRα, enabling strong receptor-mediated binding that was minimally affected by endogenous folate. When loaded with DOX or the photosensitizer methylene blue, these nanoparticles exhibited predominant uptake *via* the caveolar endocytic pathway, and *in vivo* studies confirmed preferential accumulation in HeLa tumour xenografts.^137^ In another study, paclitaxel-loaded, folate-functionalised MIPs were developed to target folate receptor-positive MDA-MB-231 breast cancer cells, resulting in markedly enhanced cytotoxicity, with an IC_50_ of 4.86 nM compared to 32.80 nM for free paclitaxel.^138^

Examples of lectins studied in oncology include galectins, which promote tumour growth and immune evasion;^139^ C-type lectins, which require Ca^2+^ for carbohydrate binding and are often overexpressed on tumour-associated immune cells;^140^ and selectins, which mediate adhesion and metastasis.^141^ Monosaccharides can be exploited for targeted drug delivery by leveraging lectin overexpression, directing nanocarriers to nutrient receptors, improving solubility, and enhancing uptake into tumour-associated immune cells.^142^ For instance, multi-responsive hydrophilic “hairy” fluorescent MIPs imprinted with sialic acid (SA) have been developed for receptor-targeted 5-fluorouracil (5-FU) delivery, enabling glutathione (GSH)-triggered intracellular release, improved tumour penetration, and simultaneous bioimaging.^143^ Similarly, carbon dot-core MIPs developed to target glucuronic acid, a substructure of hyaluronan overexpressed on cancer cells, allowed selective recognition and imaging of human cervical cancer cells.^144^

Growth factor receptors are prevalent in many cancers and associated with aggressive phenotypes and receptor-mediated endocytosis, thus making them one of the most popular targets for cancer drug delivery.^53^ MIPs developed against VEGF conjugated with quantum dots for fluorescence detection, selectively targeted VEGF-overexpressing tumours in zebrafish xenografts, exhibiting high affinity (equilibrium dissociation constant *K*_D_ of 1.56 nM) and minimal toxicity in embryos.^145^ In another recent study, bispecific MIPs engineered to target VEGF and Delta-like 4 (DLL4) achieved selective tumour accumulation in MCF-7 mouse xenografts and 68–72% tumour suppression.^146^ Furthermore, EGFR-targeted nanoMIPs imprinted against the receptor and DOX enabled selective cancer cell targeting, with a high *K*_D_ value of 3.6 nM for the receptor.^93^ Additionally, dopamine-based EGFR-imprinted MIPs demonstrated pH-responsive DOX release, enhancing delivery to receptor-overexpressing cells.^147^ In another example, MIPs targeting HER2 have been shown to increase *in vivo* DOX accumulation, enhance tumour suppression, and reduce off-target toxicity in ovarian cancer mouse models.^148^ Similarly, MIPs targeting ERα, loaded with a fluorescent dye and DOX, exhibited preferential binding to ERα (*K*_D_ = 14.7 nM) in breast cancer cell models and delivered DOX to the nucleus *via* receptor-driven uptake.^16^

Biotin, a water-soluble vitamin, acts as a cellular growth promoter and is essential for rapidly dividing cancer cells.^134^ To exploit this, biotin-functionalized core–shell MIPs were developed that specifically targeted MCF-7 breast cancer cells, demonstrated enhanced adsorption and endocytosis-mediated uptake, enabled intracellular delivery of DOX, and reduced MCF-7 cell viability from 90% to 60%.^149^ Biotin-selective MIPs have also been developed as adaptable scaffolds that bind biotinylated targeting motifs, such as a CD44-specific aptamer, enabling precise recognition of cancer cells.^150^ Interleukin receptors, a type of cytokine, stimulate the growth and invasiveness of various types of cancers, including ovarian, breast, malignant glioma, lung, colon, bladder and pancreatic carcinoma.^151^ Molecularly imprinted ‘nanotraps’ have been developed to selectively bind and sequester IL-6, effectively lowering extracellular IL-6 levels in cell models and underscoring the potential of MIPs to modulate pro-tumorigenic cytokines in cancer progression.^152^ An overview of the various receptors and cancer types is provided, along with examples that have been approved by the FDA.

#### 3.1.2. Receptor selection criteria and techniques

The targeted receptors are not confined to a single tissue type; rather, they are often expressed at higher levels in specific regions of the body.^153,154^ Their distribution can be uneven across different tissues and may also vary in density within the same tissue.^155,156^ Therefore, an ideal receptor target for imprinting should exhibit a high tumour-to-normal tissue expression ratio to minimise on-target off-tumour effects during targeted delivery.^157^ Subsequently, the targeted moieties must be accessible to the targeting agent, typically located on the cell surface and not obscured by other proteins or cellular complexes.^158^ The recognition process is also influenced by the isoform diversity of the targeted molecule. Isoform-specific expression can alter epitope availability, ligand affinity, and downstream biological responses, thereby impacting targeting efficiency.^158,159^ Receptor targeting can promote internalisation of the nanocarriers, leading to intracellular drug release, but may also temporarily remove the receptor from the cell surface, thereby limiting the binding of additional drug-carrier complexes.^158,160^ Consequently, receptor internalisation is a critical parameter when selecting targets for sustained drug delivery. For example, a study investigated the targeting potential of MIPs toward both intracellular and extracellular EGFR epitopes and demonstrated that MIPs aimed at intracellular domains can sequester the receptor, inhibit its signalling, and reduce cancer cell viability.^161^ Another factor that needs to be considered is the internalisation potential of the MIPs for drug release. The nanoparticles can enter cells through multiple endocytic mechanisms, generally *via* clathrin-mediated endocytosis. Other mechanisms include caveolae-mediated endocytosis and macropinocytosis, which are less frequent.^162^ The dominance of a particular endocytic pathway can influence intracellular trafficking and therapeutic efficacy. For instance, MIPs imprinted against EGFR have been reported to undergo endocytosis and subsequent accumulation in the cytoplasm.^93^

Computational techniques are often employed to select membrane proteins and biomarkers for the design of targeted DDSs. ML is utilised to analyse multi-omics datasets to identify protein mutations that drive tumour development and to assess their effects on protein stability and function.^163,164^ Comprehensive bioinformatics analyses of gene expression data from TCGA (The Cancer Genome Atlas), UALCAN (The University of ALabama at Birmingham CANcer data analysis Portal), TNMplot (Tumour, Normal, and Metastatic plot database), and LinkedOmics have been used to identify membrane proteins as potential targets for breast cancer therapy.^165^ In addition, protein–protein interaction networks and cell-surface proteomics are used to prioritise functionally central, accessible, and cancer-specific receptors.^166,167^ Structural prediction models such as AlphaFold2 and trRosetta can be applied to model these proteins. AlphaFold2 offers accurate structural predictions for targets lacking experimental data, thereby supporting structure-based drug design and virtual screening.^168,169^ Studies have shown that predictions with high-confidence regions are comparable to experimental structures and are valuable for computational modelling, while low-confidence regions help define domain boundaries for protein expression and functional studies.^170^ At this stage, computational modelling primarily serves as a screening and prioritisation tool for receptor selection. Subsequently, molecular docking and simulation techniques are used to characterise interactions between the proteins and the designed drugs.^163^ Once the target has been selected, polymer composition and monomer feed can be considered as described above. Naturally, surface targets are difficult to obtain a whole free protein without loss of secondary and tertiary structure, meaning that whole protein imprinting is not suitable here – targeting a region of the protein is therefore required.

### 3.2. Epitope imprinting

#### 3.2.1. Epitope imprinting selection criteria

Biological applications, such as drug delivery, require the imprinting of large biomacromolecules, like proteins. However, there are some key challenges with using these molecules as templates in molecular imprinting. First, high molecular weight can hinder mass transfer and pose difficulties for the template to diffuse out after polymerisation, though this is less of an issue with solid-phase imprinting. This diffusion limitation also slows the rebinding of the target molecules to the MIPs. While proteins and other biomolecules are more stable in aqueous solutions, certain monomers used in molecular imprinting perform best with organic solvents, limiting the choice of compatible monomers. Furthermore, proteins are heterogeneously charged and have structurally similar regions, which can lead to cross-reactivity and non-specific binding.^31,171^ As demonstrated by previous studies, MIPs synthesised against a specific protein conformation exhibit the highest affinity for that conformation during rebinding. Therefore, even minor structural changes in the protein can reduce binding affinity.^172^ These challenges are further exacerbated for membrane proteins, whose native conformation is strongly dependent on the lipid bilayer environment and is often disrupted upon removal from the membrane, leading to loss of structural integrity and binding fidelity.^173^ These limitations, alongside the need to target membrane-bound polymers, led to the use of epitopes as templates for molecular imprinting.

An epitope is a specific region of an antigen that is recognised by antibodies or other targeting molecules.^174^ These distinct protein segments act as antigenic determinants, and their defined structural features enable them to represent the entire protein in epitope-imprinting strategies. In such approaches, a short peptide fragment or structural portion of the target protein serves as a substitute or partial template for synthesising MIPs.^175^ Compared to whole proteins, epitope templates are more stable during imprinting and can be readily synthesised, even for rare targets. Since epitope-imprinted sites mimic natural antibody recognition domains, they often provide high binding specificity toward the target.^74,176^

Using a smaller molecule as a template overcomes structural and cost challenges of larger proteins while maintaining binding to the full target. The reduced size also restricts flexibility and the number of exposed functional groups, resulting in more uniform imprint cavities.^177^ Additionally, peptides are more stable than proteins because they lack tertiary and quaternary structures and can be synthesised to be soluble in a wider range of solvents, offering greater flexibility for MIP preparation. Consequently, peptide-based epitope templates improve both the reproducibility and the cost-effectiveness of MIP synthesis,^178,179^ though in some cases, reduce the affinity, likely due to steric hindrance.

An epitope can either be a linear sequence of amino acids located in the loops of a protein or be discontinuous, where the amino acids are spatially close during protein folding. Linear epitopes are generally preferred as templates in molecular imprinting because they are easier to synthesise and present a well-defined, reproducible structure, whereas discontinuous (conformational) epitopes are difficult to replicate due to the challenge of reproducing their native three-dimensional spatial arrangement.^74,177^

While linear epitopes offer practical advantages as templates for molecular imprinting, concerns remain regarding whether recognition of a short linear peptide sequence can translate to binding of the same region in the folded native protein. Factors such as protein conformation and steric hindrance may limit epitope accessibility. However, several studies have demonstrated that epitope-imprinted MIPs can successfully recognise the corresponding full-length receptor in biological systems. For example, nanoMIPs imprinted against short peptide epitopes derived from cancer receptors have shown selective binding to receptor-expressing cells and receptor-mediated endocytosis, indicating that recognition of the linear peptide template can translate to interaction with the native membrane-bound protein.^16^ In addition, conformational epitopes can also be employed when more accurate protein recognition is required in complex physiological environments. Unlike linear epitopes, conformational epitopes consist of discontinuous amino acid residues that become spatially close after protein folding, thereby better reflecting the protein's native three-dimensional structure. Imprinting strategies based on such epitopes may therefore improve the specificity and binding affinity of MIPs.^180^ However, their practical application can be limited by structural instability or susceptibility to degradation outside the native protein environment, thereby hindering accurate imprinting. Furthermore, a direct validation of MIP-protein recognition has been demonstrated using ELISA-type assays.^181,182^ For instance, MIP-based pseudo-ELISA assays developed for procalcitonin detection achieved limits of detection of 3.8 ng mL^−1^ in buffer and 4–6 ng mL^−1^ in plasma, demonstrating analytical performance comparable to its conventional antibody-based ELISA assays.^181^

The concept of epitope imprinting involves imprinting a portion of the protein, rather than the entire molecule, offering a more economical and compatible alternative with various monomers and solvents.^179^ The concept of epitope imprinting was first proposed by Rachkov *et al.*, who utilised a tetrapeptide derived from oxytocin instead of the entire peptide in MIP synthesis to selectively bind oxytocin in an aqueous environment.^175^ Subsequently, a study by Tai *et al.* was the first to use an epitope for protein recognition, employing a 15-mer peptide for imprinting to recognise the NS1 protein of dengue virus.^183^

The selected epitope is typically a surface-based segment of the targeted protein that enables specific binding. Often, C- or N-terminal epitopes are chosen as templates; however, N-terminal is less favourable due to its higher susceptibility to post-translational modifications, which can alter the structure of the peptide and interfere with the imprinting.^74^ Choosing a linear epitope has several advantages: it is cost-effective, its binding sites are accessible on the protein surface, its position within the protein simplifies selection, and it can be readily modified to meet synthesis requirements.^184^

Another important criterion in epitope selection is its length. While too-long peptides can have complicated structures, peptides that are too short can lead to ineffective imprinting and low binding affinity of the MIP.^184^ One study demonstrated that epitope length affects MIP performance, with a 14-amino-acid peptide exhibiting the highest binding affinity. However, it was emphasised that factors beyond epitope length and conformation significantly impact MIP performance.^185^ Another comprehensive study analysed the impact of epitope length by comparing three epitopes with 9, 12 and 15 amino acids, derived from the C-terminal of human serum albumin. The 12-mer peptide yielded the best imprinting factor, characterised by the highest specificity and affinity, underscoring the importance of considering peptide length during epitope selection.^186^ Additionally, it has been observed that other factors, including hydrophobicity and solvent compatibility, together with a peptide length of less than 16 amino acids, are required for optimal MIP binding.^187^ It is acknowledged, though, that this is target-dependent, meaning there are no easy selection criteria.

#### 3.2.2. Epitope selection techniques

**Fig. 1:**
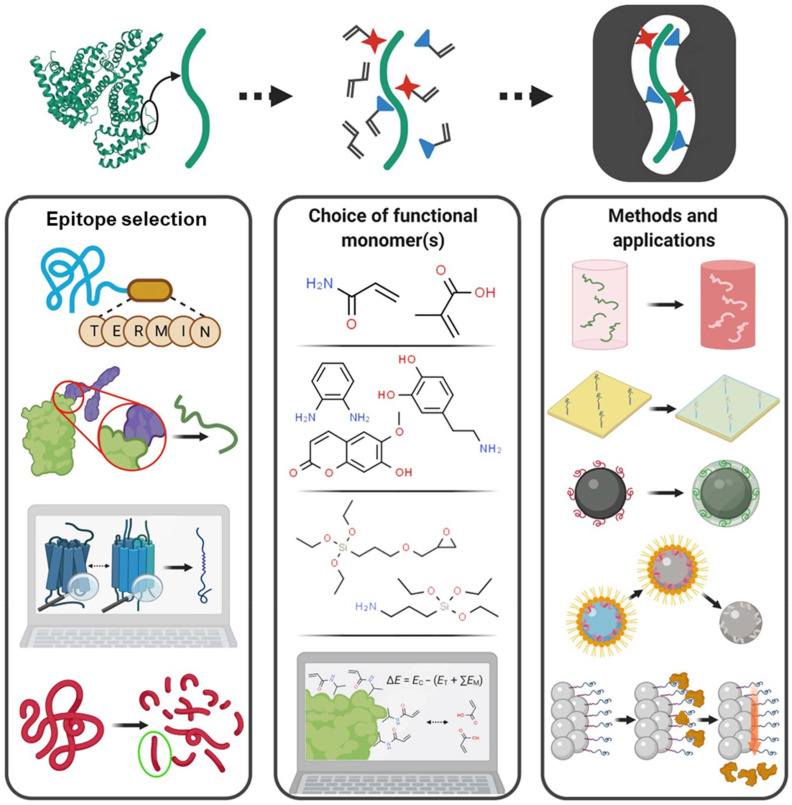
Major steps involved in the epitope imprinting process. Each step offers multiple design options that must be carefully selected to optimise MIP performance for the intended application. This figure has been reproduced from ref. 184 with permission from the American Association for the Advancement of Science, 2026.

The process of selecting an epitope for an antibody synthesis is known as epitope mapping.^174^ Several methods exist for this; the most common is X-ray crystallography of the antibody–antigen complex. Other approaches include NMR or evaluating antibody binding to different protein fragments. However, these techniques are often expensive and time-consuming. To address these limitations, *in silico* methods such as machine learning and immunoinformatic tools are being used. Factors commonly considered include selecting C- and N-terminal regions, peptide lengths of 8–20 amino acids, and preference for surface-exposed regions.^188^ An identical approach can be used to select an epitope for molecular imprinting. Commonly, the procedure for choosing an epitope for imprinting is based on the above-discussed criteria. The epitope imprinting process is illustrated in Fig. 1. The C- or N-terminal of a protein with an appropriate peptide length is often chosen.^189,190^ Another common approach is to identify a region of the protein (or antigen) to which the antibody binds and use that sequence as a template, or guidance from biological epitope-paratope complexes. This strategy uses known, commercially available antibodies to obtain the peptide sequence and to synthesise the artificial antibodies as MIPs.^66,93^ This approach allows biologically validated recognition motifs to be directly translated into synthetic imprinting systems.

Other chemical-based methods are also used for selecting the epitope. One such approach involves immobilising the target protein on a solid support and then digesting it enzymatically. This results in the selective retention of surface-accessible peptide fragments, which can then be used directly as templates.^191^ This concept was employed using acetyl cholinesterase as the model protein, which was first imprinted onto the polymer. After enzymatic digestion of the protein without removal from the polymer, the remaining polymer-bound sequences are eluted and identified by mass spectrometry. In this case, seven peptide sequences were identified, four of which were consistent with the known literature epitopes.^192^

Another study employed a similar concept-based method, snapshot imprinting, for biomarker discovery and epitope selection in complex systems. In this approach, MIPs were synthesised in the presence of whole cells, enabling the polymers to ‘record’ the exposed surface proteins. Subsequent trypsin digestion and mass spectrometric analysis identified three senescence-specific proteins, which can be utilised for the development of targeted personalised medicine for cellular ageing.^193^ This strategy demonstrates the potential of MIPs to identify biologically relevant epitopes directly from complex biological environments.

A common strategy for epitope selection is based on computational or *in silico* modelling. This approach has gained attention for its cost-effectiveness and ability to reduce experimental burden. Both epitope templates and suitable functional monomers can be selected computationally, reducing monomer and solvent consumption and minimising waste generation.^59,188^ This method involves screening monomer libraries to select those with the most favourable interactions and identifying peptide epitopes that exhibit stable conformations. For example, MD simulations have been used to analyse the stability of peptide segments derived from neuron-specific enolase (NSE) and to identify regions that remain stable under physiological conditions.^194^

Bioinformatic resources such as the Immune Epitope Database and Analysis Resource (IEDB) can also support epitope identification. The UniProt Knowledgebase (UniProtKB) provides curated amino acid sequences of target proteins, while tools available through the ExPASy portal can simulate enzymatic digestion to generate peptide fragments with suitable length and orientation. The peptides can then be analysed using BLAST to assess sequence specificity and surface relevance.^179,188^ In one study, BLAST analysis was used to identify biomarkers associated with idiopathic pulmonary fibrosis, leading to the selection of two surface-accessible epitopes. The resulting sensor successfully detected the epitopes in complex biological media.^195^

A related *in silico* strategy, termed fingerprint imprinting, integrates computational proteomics with molecular imprinting for rational MIPs design. The target protein is virtually cleaved into peptide fragments, screened against UniProtKB, and unique peptides are synthesised as imprinting templates. This approach was demonstrated using NT-proBNP, yielding MIPs with high selectivity and binding capacity.^196^ By combining experimental techniques and computational approaches, epitope selection is becoming increasingly streamlined, reinforcing the role of epitope-based MIPs in targeted drug delivery applications. Once selected, the epitope can be used as a free (in solution) or fixed (solid-phase) template for imprinting with a degree of simplicity.

## 4. MIP-based case studies in cancer therapy

As discussed in the previous sections, MIPs have several advantages that make them suitable candidates for biomedical applications. They are suitable for drug delivery due to their high loading capacity, high stability, control over crosslinking and, of course, the high affinity, which makes it easier to target affected sites.^197^ The first-ever demonstration of MIPs for drug-delivery applications was reported in 1998. The polymer composed of methacrylic acid (MAA) and ethylene glycol dimethacrylate (EGDMA) could selectively distinguish theophylline from its structural analogue, caffeine. Moreover, it exhibited a sustained release profile for theophylline, demonstrating the potential of MIPs for controlled drug delivery applications.^198^ Since then, the field has advanced remarkably, with MIPs-based DDSs evolving from simple recognition polymers to nanoscale carriers for the controlled delivery of anticancer, antibiotics, antivirals, anti-inflammatory, and several other drugs.^197^

### 4.1. Structural and imprinting design of MIP-based DDSs

A significant portion of drug delivery research focuses on cancer, owing to the limitations of traditional chemotherapy. Accordingly, MIPs have been engineered to address these challenges by enabling the controlled release of anticancer agents. The mechanism of drug delivery with MIPs can be designed through different routes. One common strategy involves engineering MIPs using tumour cell surface markers as template molecules to achieve targeted delivery, allowing the nanocarriers to recognise and bind specifically to cancer cells, as highlighted above.^137,199,200^ Here, the developed MIPs are generically loaded with the carrier therapeutic. Another technique involves creating cavities using the drug itself as the template. This approach provides a high drug-loading capacity and facilitates controlled, prolonged release by utilising the imprinted cavities as reservoirs for drug molecules, though it does not provide targeting.^201,202^ In contrast to receptor-mediated endocytosis as an internalisation method (as seen with surface marker templates), drug-imprinted MIPs are generally internalised through non-specific or passive mechanisms, such as adsorptive endocytosis or diffusion-driven uptake, and are primarily governed by physicochemical properties, including particle size, surface charge, and hydrophobicity.^120,203^ Additionally, stimuli-responsive release mechanisms, along with combinational therapies, are employed to achieve controlled drug release, as discussed in later sections.

Apart from the imprinting strategy, the structural architecture of MIPs-based nanocarriers also plays a vital role in influencing their drug delivery performance. Different structural configurations have been developed to optimise drug loading, protection, and release profiles. Core–shell imprinting (coating the MIP around an inert core) positions binding sites near the MIP surface, enabling faster target binding and release while reducing mass-transfer resistance. Such behaviour has been exemplified in photoluminescent core–shell nanocarriers, where gelatin quantum dots coated with a methotrexate-imprinted MIP shell enabled selective drug recognition and sustained release.^204^ In another study, metal–organic framework (MOF)-based MIPs were engineered as core–shell nanocarriers, integrating the high drug-loading capacity of a Cu-MOF core with a DOX-imprinted polymer shell, resulting in pH-responsive and sustained drug release under tumour-relevant conditions.^205^ Magnetic cores are often used in targeted drug delivery to enable stimuli-responsive drug release, as discussed in detail in later sections.

In addition to core–shell systems, capsule-like nanoparticles have also been developed to achieve high drug loading and targeted delivery. For example, multi-responsive fluorescent MIP nanocapsules meeting the 2R2SP (balancing drug retention and on-site release, switching between stealthy and adhesive surface states, and enabling deep tumour penetration beyond blood vessels) requirements demonstrated prolonged circulation, targeted tumour accumulation, high loading capacity, rapid intracellular release, and bioimaging potential owing to their SA-imprinted shell and flexible semi-hollow structure.^143^ Similarly, polydopamine-based capsule-like nanoMIPs were developed for synergistic chemo-photothermal cancer therapy, where EGFR served as the template and ZIF-8@DOX as the sacrificial core, enabling targeted and controlled drug delivery along with pH- and NIR-responsive behaviour.^147^ Overall, these studies highlight that both the imprinting strategy and the structural design of MIPs-based nanocarriers are critical for achieving targeted, controlled, and stimuli-responsive drug delivery.

### 4.2. Stimuli-responsive DDSs

Beyond the material composition and target, drug release from any nanoparticle-based DDSs is also dependent on the stimuli that initiate it. This release can be either internal (pH, redox potential, enzymes) or external stimuli-based (temperature, light, magnetic field).^206^ One of the advantages of using MIPs as drug delivery vehicles is that they can be tuned to release drugs across different delivery routes, as discussed in Table 3.

**Table 3:** MIP-based stimuli-responsive drug delivery systems with fabrication method and associated studies

Stimulus type	Mechanism	Fabrication approach	Studies
pH-Responsive	Acidic tumour pH weakens polymer–drug interactions	Boronate-affinity imprinting; polymerisation with borate ester bonds	DFCR & SA MIPs;^208^ CAPE MIPs;^209^ 5-FU colon-specific MIPs^165^
Redox-responsive	Disulfide bond cleavage in a high-GSH environment	Incorporation of disulfide-containing crosslinkers (BACy, DSDMA)	BACy/DSDMA MIPs;^211,212^ organosilica MIPs for DOX + chlorin e6^213^
Magnetic field	AMF disrupts hydrogen bonds & induces hyperthermia	Core–shell magnetic nanoparticles coated with imprinted polymer	DOX magnetic MIPs;^169^ 5-FU magnetic MIPs;^170^ glucose-crosslinked magnetic MIPs^64^
Light-responsive	NIR or visible light triggers structural changes	Integration of graphene quantum dots or azobenzene monomers	DOX release *via* NIR (808 nm);^216^ azobenzene-MIPs reversible with blue/red light^217^
Temperature-responsive	LCST-driven hydrophilic/hydrophobic switch	NIPAM-based polymer grafted on natural polysaccharide or magnetic seeds	NIPAM-MIPs on konjac glucomannan;^220^ Fe_3_O_4_ thermal seeds coated with MIPs^221^
Enzyme-responsive	Template enzyme binding induces nanoparticle disassembly	Chitosan-phthalate polymer imprinting with enzyme templates	Lysozyme/α-glucosidase-responsive MIPs^222^

The most used internal stimuli in DDSs are pH-based drug release, as the tumours are known to exhibit an acidic pH compared to physiological pH.^207^ For example, boronate-affinity MIPs imprinted with 5′-Deoxy-5-fluorocytidine (DFCR) and SA exhibited dual functionality, where acidic conditions enhanced tumour targeting *via* strengthened boronate-SA interactions while weakening boronate-DFCR binding to trigger drug release, enabling pH-responsive delivery.^208^ Similarly, a pH-responsive MIP carrier for capecitabine (CAPE) was prepared, where cleavage of borate ester bonds under acidic tumour-like conditions triggered controlled drug release, leading to effective inhibition of breast cancer cells.^209^ Exploiting a different mechanism, pH-responsive MIPs for 5-FU were developed for colorectal cancer, where protonation-driven weakening of polymer–drug interactions enabled colon-specific release at pH 7.4. This minimised premature release of 5-FU and improved therapeutic efficiency.^210^

Other stimuli, such as redox responsiveness, have also been exploited for controlled drug release by incorporating disulfide-containing crosslinkers, including *N*,*N*′-bisacrylylcystamine (BACy) and bis(2-methacryloyloxyethyl) disulfide (DSDMA). These designs leverage the elevated GSH levels in the tumour microenvironment, where cleavage of disulfide bonds triggers drug release from the nanocarriers. As a result, the drug remains stable under normal physiological conditions but is selectively released within tumour tissues.^211,212^ Interestingly, these nanoMIPs exhibit lower *K*_D_s (µM) than solid-phase systems risking off-target binding. Similarly, organosilica-based biodegradable MIPs have been engineered to respond to both pH and redox stimuli, where the MIP shell enables selective cancer cell targeting, while disulfide bond cleavage in the organosilica core induces the release of DOX and the photosensitizer chlorin e6, facilitating combined chemotherapy and photodynamic therapy.^213^

Another approach combined pH-responsive release with external magnetic fields, which disrupted hydrogen bonds between DOX and the MIPs, and included local hyperthermia to enhance anticancer efficacy.^214^ Core–shell magnetic MIPs have been shown to achieve extended drug release of 5-FU for up to 200 h, compared to 57 h for non-imprinted controls.^215^ Similarly, biodegradable MIPs crosslinked with glucose were developed for targeted delivery of docetaxel, where magnetic properties enabled both precise tumour localisation and controlled drug release.^106^ Some DDSs are also engineered for photo-responsive release. For instance, graphene quantum dots-MIPs triggered the release of DOX upon 808 nm near-infrared (NIR) light, weakening the interactions between the drug and the polymer matrix. Additionally, the release kinetics partially followed a zero-order model, indicating sustained and controlled drug release.^216^ In another case, visible-light-responsive MIPs bearing azobenzene undergo a conformational change from trans to *cis* with 400 nm blue light and revert to the trans conformation with 630 nm red light. These reversible structural properties allowed controlled drug loading and release.^217^

*N*-Isopropylacrylamide (NIPAM) is frequently employed in the design of thermoresponsive MIPs due to its lower critical solution temperature (LCST) of 32 °C, which is close to human body temperature. Of note, NIPAM is also a common monomer used in solid-phase imprinting to facilitate thermal polymerisation. Below the LCST, the polymer exhibits hydrophilic behaviour, facilitating drug loading, whereas above this temperature, it becomes hydrophobic, triggering drug release.^218,219^ Exploiting this property, a biocompatible thermoresponsive NIPAM-MIPs-based DDS was developed by grafting the polymer onto konjac glucomannan, a natural polysaccharide. This system was capable of selectively binding and releasing 5-FU in response to temperature changes while exhibiting high selectivity and stability around the LCST.^220^ In another approach, Fe_3_O_4_ magnetic thermal seeds coated with thermoresponsive MIPs were developed for methotrexate (MTX) delivery. Exposure to an alternating current magnetic field (AMF) rapidly increased the MIP temperature, triggering a release of up to 80% of the loaded drug due to the weakening of hydrogen bonds.^221^

Alongside other stimuli, enzyme-responsive MIPs have been developed, in which specific, non-catalytic binding by template enzymes, such as lysozyme or α-glucosidase, induces structural changes in chitosan-phthalate nanoparticles. This triggered the selective disassembly of nanoparticles and on-demand drug release, achieving approximately 90% compared to 11% in non-imprinted controls, driven by enzyme-specific binding.^222^ Thus, the combination of stimulus-responsiveness and selectivity in MIPs leads to the development of controlled-release systems that can significantly enhance therapeutic effects while reducing systemic toxicity.

### 4.3. Hybrid MIPs and MIPs with further complexity

While traditional MIPs are valuable in nanomedicine due to their high selectivity, their properties can be further enhanced by integrating them with complementary nanoparticles, biological molecules, or inorganic functional components. Such hybrid systems improve the selectivity, precision, stimuli-responsiveness, versatility, and overall efficacy of the MIPs.

**Fig. 2:**
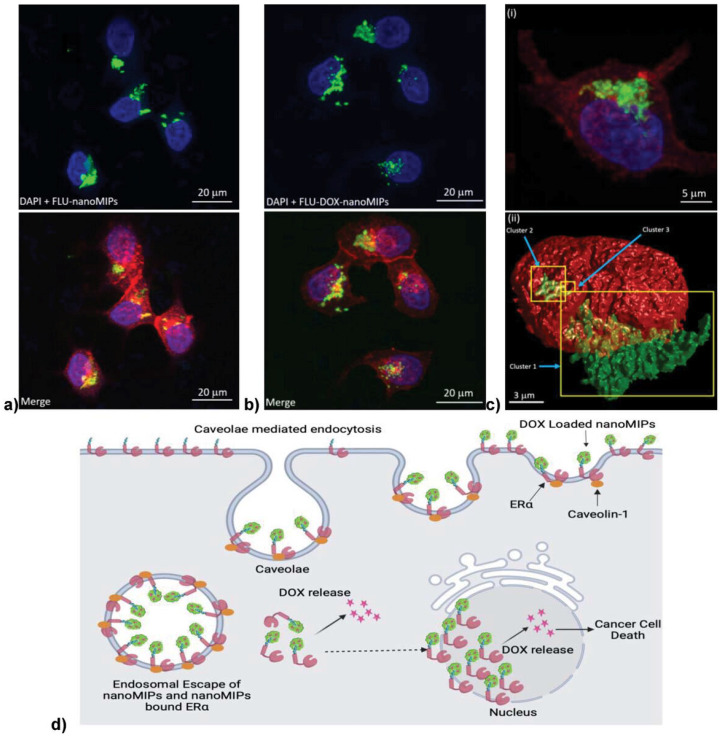
Confocal laser scanning microscopy (CLSM, 63×) revealed efficient uptake of (a) fluorescent nanoMIPs (FLU-nanoMIPs) and (b) drug-loaded variants (FLU-DOX-nanoMIPs) by ERα-positive MCF-7 breast cancer cells after 24 h incubation at 37 °C, (c) (i) 3D reconstructions confirmed intracellular accumulation and partial nuclear localisation, with green fluorescence (nanoMIPs) colocalising with blue-stained nuclei (DAPI) and red-stained membranes (WGA-Alexa Fluor 594), (ii) yellow regions in the 3D render indicate nanoMIP clusters within the nucleus, suggesting receptor-mediated endocytosis followed by nuclear translocation (d) schematic illustration of caveolae-mediated uptake *via* ERα, followed by subsequent cytoplasmic trafficking and DOX release in both the cytoplasm and nucleus, thereby enabling enhanced therapeutic effects.^16^

Within the framework of hybrid MIPs, double-imprinted polymers, or dual-template MIPs, are prepared using multiple templates during polymerisation, enabling them to simultaneously recognise, bind, load, detect, analyse, and separate multiple analytes.^223^ A specific form of double imprinting, mostly used in DDSs, involves using a protein or epitope as a recognition template and a drug as the second imprint, which remains loaded on the MIP. Canfarotta *et al.* employed an EGFR epitope alongside DOX as templates. The resulting MIPs specifically targeted the cancer cells overexpressing EGFR and simultaneously released DOX at the tumour site.^93^ Another study on double imprinting, targeting Erα and imprinted with DOX, resulted in 80% cytotoxicity in ERα-positive cancer cells. These nanoMIPs enabled receptor-mediated endocytosis and nuclear drug delivery, as shown in Fig. 2.^16^

Similar systems have integrated breast cancer cell membrane proteins with chemotherapeutic agents to create fluorescent MIPs capable of effective tumour suppression both *in vitro* and *in vivo.*^224^ Overall, double imprinting is an emerging and promising strategy in the field of drug delivery, though a broad set of questions remains around the compromises in affinity and selectivity that arise from dual-target imprinting (template-template interactions and compatibility) and capacity issues. Despite this, continued research in this area is required as it holds great potential.

Inorganic moieties, such as magnetic nanoparticles, metal–organic frameworks, silica-based nanoparticles, and quantum dots, can be integrated with MIPs to enhance their drug-delivery functionality.

Conjugating with magnetic nanoparticles not only simplifies purification during synthesis but also enables targeted delivery and helps mitigate rapid metabolism associated with chemotherapeutics. For example, core–shell Fe_3_O_4_-MIPs systems for 6-mercaptopurine combined magnetic targeting and hyperthermia with selective binding and controlled drug release, where the inorganic core enables stimuli-responsive delivery and the organic MIPs shell provides high specificity and drug-loading efficiency.^225^ These magnetic nanoparticles are among the most used functional conjugates in MIPs, enabling a wide range of versatile applications. In another study, core–shell nanocarriers were constructed with a magnetic metal–graphene oxide core and a dopamine-based MIP shell imprinted against CEA, a cancer biomarker. This system exhibited dual targeting through magnetic guidance and molecular recognition, along with pH-responsive drug release, leading to improved anticancer efficacy.^226^ Additional magnetic MIPs-based strategies have been reported and are addressed elsewhere in this review.

Additionally, metallic components, such as metal–organic frameworks (MOFs), are widely used in DDSs. MOFs are another class of hybrid materials characterised by high surface areas and tunable porosity, making them excellent candidates for drug delivery.^227^ Integrating MOFs with MIPs combines the selective recognition of MIPs with the high loading and controlled release capacity of MOFs. For example, a recent study on Cu-MOF-based hollow MIPs reported exceptional DOX-loading efficiency (81.1%) and pH responsiveness and showed strong cytotoxicity against MCF-7 cells with an IC_50_ of 2 µg mL^−1^, whereas free DOX required higher concentrations to elicit comparable effects.^98^ Another study employing a zirconium-based MOF (UIO-66) integrated with deep-eutectic solvent-assisted dual-templated MIPs against DOX and phycocyanin demonstrated improved hydrophilicity, high drug-loading capacity, and controlled drug release.^228^ In addition to these, MOFs such as ZIF-8, MIL-101(Fe), and UiO-66-NH_2_ have also been employed in MIP-based DDSs, demonstrating high drug-loading capacity, controlled release, and stimulus-responsive targeting.^104^

Silica-based nanoparticles serve as functional scaffolds for MIPs-based targeted drug delivery, offering mechanical stability and high payload capacity. Among these, mesoporous silica nanoparticles are widely used as substrates for developing these DDSs, providing high surface area, structured surfaces, mechanical robustness, and biocompatibility.^199,229^ Additionally, siloxanes have been incorporated into MIPs chemistry to enhance flexibility, durability, ease of functionalization, and imprinting efficiency.^104,230,231^

Another class of hybrid nanomaterials integrates quantum dots with MIPs, combining selective molecular recognition with photoluminescence to enable simultaneous drug delivery and bioimaging. For this purpose, carbon quantum dots,^204^ graphene quantum dots,^216,232^ and fluorescent silica nanoparticles (FSiO_2_-NPs)^224,233^ have been employed as functional cores. In a recent study, dual-template MIPs developed against the HER2 peptide (overexpressed in several tumours) and DOX utilised FSiO_2_-NPs as the core for targeted DOX delivery, enabling both diagnostic imaging and therapeutic functionality.^233^ Similarly, a dual-imprinted DDS based on FSiO_2_-NP targeting the P32 membrane protein epitope enabled real-time cellular tracking *via* intrinsic fluorescence, with confocal and dual-colour imaging confirming DOX release and nuclear accumulation in cancer cells.^224,233^

Besides carbon and graphene quantum dots, other carbon-based materials, such as graphene oxide (GO) and carbon nanotubes (CNTs), have also been combined with MIPs for drug delivery applications. Both GO and CNTs are employed in the design of DDSs due to their high surface area, ease of functionalization, strong drug-loading capacity, π–π interactions, and structural stability.^234^ For instance, a vinyl-functionalized GO surface was coated with a curcumin-imprinted polymer, forming a pH-responsive nanocomposite for selective binding and release of curcumin, with enhanced drug loading *via* β-cyclodextrin inclusion.^235^ Subsequently, CNTs combined with POSS enhanced the imprinting efficiency of MIPs, improved the pore structure and surface area, provided superior controlled *in vitro* release of gallic acid, and higher *in vivo* plasma concentrations, achieving a maximum AUC_0–9_ of 544.73 ng h mL^−1^ compared to lower values for control MIPs.^236^ In another study, liquid-crystalline MIPs-based nanocarriers were constructed on CNT supports *via* surface imprinting for the oral delivery of levofloxacin. The system achieved zero-order release, prolonged gastric retention, and markedly enhanced bioavailability, demonstrating the potential of MIPs-CNT hybrids for controlled drug delivery.^237^

Other functional materials, such as aptamers, have been integrated with MIPs to leverage their molecular recognition capabilities, primarily in sensing. Aptamer-MIPs hybrids have been employed in the detection of proteins, enzymes, genes, pathogens, and various other analytes, including pesticides, antibiotics, and explosives.^238–240^ Aptamers, which are single-stranded DNA or RNA molecules, have also gained attention in drug delivery, as they can modulate biological targets with high specificity and enable targeted delivery.^241^ However, MIPs offer greater stability, improving affinity and are less susceptible to degradation.^238^ Unlike base aptamers, those entrapped within a MIP scaffold can be exposed to high temperatures, DNAase, RNAase, pH 2–12 and organics without loss of action.^242,243^ Despite this potential, studies on aptamer-MIPs systems for drug delivery are scarce, and the field remains underexplored, presenting significant opportunities for future research.

### 4.4. Combination therapies

The effectiveness of cancer treatment can be enhanced by combining therapeutic strategies that act in a complementary manner to overcome multidrug resistance, minimise systemic toxicity, and achieve precise site-specific targeting. MIPs have emerged as a versatile platform not only for drug delivery but also for integration with other therapeutic modalities such as photothermal therapy (PTT), photodynamic therapy (PDT), magnetic hyperthermia, and imaging-guided theranostics, as summarised in Table 4.

**Table 4:** Combinational therapies based on MIPs strategies

Therapeutic modality	Mechanism/key advantage	MIP functionalization strategy	Example/evidence
Chemotherapy + PTT	Converts light to heat → hyperthermia kills cancer cells; synergistic with chemo to reduce drug dose and regrowth	Incorporated photothermal nanoparticles (*e.g.*, Fe_3_O_4_); used photothermal monomers, loaded photothermal agents	Iron-oxide core + DOX-imprinted + epitope-imprinted outer layer → NIR-triggered ablation;^244^ Polydopamine/ZIF-8 nanocarriers for EGFR targeting^147^
PTT Sensitization	Overcomes heat tolerance by inhibiting HSP expression	Imprinted against metabolic enzymes (*e.g.*, hexokinase) to induce tumour starvation	Hexokinase-epitope MIPs reduce glucose metabolism → enhance PTT^110^
Chemotherapy + PDT	Photosensitizer generates ROS under light → selective cell death	Embed photosensitizers in MOF or MIPs layer	Dual-gated MOF system with epitope-imprinted ZIF-8 and DNA gate for chemo + PDT;^111^ black TiO_2_ MIP nanocomposites for 5-FU delivery + PTT + PDT^245^
Magnetic hyperthermia	AMF triggers local heating → controlled drug release	Magnetic nanoparticle core + imprinted polymer shell	γ-Fe_2_O_3_@DOX-MIPs enable AMF-triggered DOX release;^246^ Comparative study: MIPs *vs.* nanogels for AMF-triggered release^247^
Immunotherapy/TME modulation	Reprograms immunosuppressive microenvironment; enhances drug penetration	Tryptase-imprinted MIPs reduce stromal barriers; PD-L1 peptide imprinting blocks PD-1/PD-L1; sialic acid imprinting promotes TAM phagocytosis	Tryptase-targeting MIPs improve DOX/LIP penetration;^248^ PD-L1-imprinted CaCO_3_ nanospheres + aptamer for immune checkpoint blockade;^249^ Magnetic core + SA-imprinted shell for TAM activation^250^

PTT employs photothermal agents that convert absorbed light energy into localised heat, raising the temperature of tumour tissues and inducing cancer cell death through hyperthermia. When combined with chemotherapy, PTT can reduce the likelihood of residual tumour regrowth while achieving comparable therapeutic effects with lower chemotherapeutic doses.^251^ MIPs can be functionalized for PTT by incorporating photothermal nanoparticles as cores,^244^ using photothermal monomers,^147^ or loading photothermal agents.^53^ For example, a MIPs-based nanoplatform was developed for combinational therapy with an iron-oxide nanoparticle core, an inner DOX-imprinted layer, and an outer P32 epitope-imprinted layer. This system enabled tumour-specific targeting, pH-responsive drug release, and NIR-triggered photothermal ablation, demonstrating its potential for synergistic chemo-photothermal therapy.^244^ Similarly, nanocarriers combining imprinted polydopamine and ZIF-8 (zeolitic imidazolate framework-8) were developed against EGFR, for targeted delivery, controlled DOX release, and effective tumour ablation, enabling a synergistic chemo-photothermal therapeutic effect.^147^ MIPs have also been employed to overcome the heat tolerance of tumour cells, which arises from heat shock proteins and limits the efficiency of PTT. For instance, MIPs imprinted against hexokinase epitopes inhibited glucose metabolism to induce tumour starvation, thereby reducing HSPs expression and sensitising tumours to hyperthermia, ultimately enhancing PTT efficacy.^110^

Another therapeutic technique, PDT, uses a photosensitising agent activated by light to produce reactive oxygen species that selectively destroy target cells.^252^ In a recent study, a dual-gated, epitope-imprinted MOF system was developed for targeted cancer therapy, dual-drug chemotherapy, and PDT. In this system, DNA bound to the MOF served as the first gate and the carrier, while epitope-imprinted ZIF-8 films formed a second gate, providing selectivity, protection of DNA from degradation, and resistance to plasma phosphates. Embedded photosensitizers in the MOF enabled PDT by generating cytotoxic singlet oxygen upon light irradiation, complementing the chemotherapeutic drugs for synergistic treatment.^111^ A multifunctional platform was developed that combined drug delivery, PTT and PDT as MIPs-coated black titanium dioxide nanocomposites. Here, the thermosensitive and pH-sensitive MIPs layer allowed selective 5-FU loading and controlled release, while the black titanium dioxide core served as both a photothermal agent, converting light into heat, and a photodynamic agent, generating ROS under light irradiation.^245^

As discussed above, MIPs are often functionalised with magnetic nanoparticle cores for guided drug delivery, magnetic separation, and magnetic hyperthermia-assisted chemotherapy. AMF have been used to trigger drug release from such magnetic MIPs. For example, γ-Fe_2_O_3_@DOX-MIPs with a poly(acrylamide) imprinted shell around magnetic cores enabled precise DOX release under AMF, locally disrupting hydrogen bonds at the polymer–drug interface without bulk heating, allowing spatially controlled chemotherapy activation.^246^ A comparative study evaluated magnetic nanogels and magnetic MIPs for AMF-triggered DOX release. In nanogels, release was driven by polymer conformation changes induced by local MNP heating, whereas in MIPs, it resulted from the disruption of hydrogen bonds between DOX and the polymer. Under AMF, MIPs released approximately 60% of DOX compared to 10% without AMF, while nanogels released 45% *versus* 24%. Both systems showed efficient cellular uptake and enhanced cytotoxicity in PC-3 cells, demonstrating the potential of localised magnetic hyperthermia for targeted chemotherapy.^247^

Beyond conventional drug delivery, MIPs can improve therapeutic outcomes by reprogramming the immunosuppressive tumour microenvironment and enhancing both drug penetration and bioavailability. Building on this concept, tryptase-targeting MIPs were developed to modulate tumour-associated fibroblasts and promote intra-tumoral delivery of doxorubicin liposomes (DOX/LIP). These nanoparticles bind selectively to tryptase, exert inhibitory effects, and help reduce stromal barriers, thereby facilitating deeper penetration of drugs.^248^ In another study, a dual-targeting system was developed by imprinting a PD-L1 peptide on a CaCO_3_ nanosphere core, followed by aptamer modification. The system specifically binds PD-L1, blocks PD-1/PD-L1 interactions, reactivates T cells, and inhibits tumour growth, with a detection limit as low as 0.003 mg mL^−1^.^249^ In a complementary strategy, a magnetic core was combined with an SA-imprinted shell to engineer reprogrammed tumour-associated macrophages (TAMs). Guided to the tumour by an external magnet, the nanoparticles bound SA on cancer cells, tagged them for phagocytosis, and promoted M1 macrophage polarisation, which boosted TAM-mediated antitumor immunity without affecting normal tissue.^250^ Overall, these advances highlight the potential of MIPs as multifunctional therapeutic platforms for more precise, effective, and synergistic cancer therapy.

## 5. Challenges and translational potential

Biocompatibility is a critical consideration for nanomaterials, particularly for biomedical applications such as drug delivery. A comprehensive *in vitro* study evaluating the biocompatibility and cellular internalisation of MIPs across HaCaT (human keratinocytes), MEFs (mouse fibroblasts), HT1080 (human fibrosarcoma), and macrophages demonstrated minimal cytotoxicity, negligible macrophage activation, and particle uptake that was dependent on surface chemistry and protein corona formation.^253^ Another study investigated the biodistribution, clearance, and cytotoxicity of MIPs following intravenous and oral administration, showing that nanoMIPs distributed to all major organs and were primarily eliminated as whole particles *via* urine and faeces. At lower doses (100 µg mL^−1^), minimal histological alterations were observed, whereas higher doses (200 µg mL^−1^) elicited mild inflammatory responses and minor tissue changes, highlighting the importance of dose-dependent safety assessment.^254^ In the context of drug delivery, loaded MIPs have demonstrated significantly higher therapeutic impact on targeted cancer cells compared with normal cells. For example, pH/GSH-responsive CD73 epitope-imprinted MIPs exhibited less than 7% drug leakage over 96 h under physiological conditions, while releasing more than 90% of the payload in tumour microenvironment conditions. Moreover, their uptake was 4.5-fold higher in CD73-overexpressing 4T1 cancer cells compared with CD73-low TC-1 cells.^255^ In another study, the safety of MIPs was evaluated using an MTT assay on HepG2 cells, which showed that cell viability increased with increasing polymer concentration. Specifically, survival increased from approximately 60% at 0.3 mg L^−1^ to 83% at 100 mg L^−1^ after 72 h, confirming the non-toxic nature of the carrier designed for docetaxel delivery.^106^

However, since clinical trials have not yet been conducted with MIPs and there are limited literature studies evaluating the *in vivo* interactions of these materials in animal models, our understanding of the biodistribution, (long-term) cytotoxicity and clearance of MIPs for *in vivo* applications is underdeveloped. Standardised testing procedures need to be implemented, as batch-to-batch variation can occur due to a lack of control during polymerisation or variations in purification procedures, leading to heterogeneity in functionalities, chemical composition, molecular weight, and particle dimensions. Moreover, size, surface (and internal) chemistry, and mechanical characteristics (unique to each MIPs) have a critical influence on biocompatibility.^73^ Researchers can choose between designing a degradable MIPs-based system or pursuing avenues in which the MIPs are cleared as intact particles. Both routes require detailed studies on the degradation of MIPs under physiologically relevant conditions, material stability, and investigation of potential leaching of monomers and other chemicals. Critically, interpreting such biological outcomes is further complicated by the fact that the final polymer composition often deviates from the feedstock formulation; key physicochemical parameters, including particle mass, molecular weight distribution, cross-linker density, and charge, strongly influence *in vivo* behaviour yet remain difficult to measure and control accurately.

It has been reported that the biocompatibility of MIPs can be enhanced by carefully selecting non-toxic monomers, crosslinkers, and initiators. For instance, naturally derived biodegradable polymers, including protein-based polymers such as albumin, gelatin, and collagen, as well as polysaccharides like chitosan, dextran, alginate, hyaluronic acid, and cyclodextrins, can be utilised to produce MIPs.^37^ Autoclaving of MIPs without loss of functionality is possible due to the robustness of the material and can help extend the shelf life of MIPs and minimise the risk of bacterial contamination during storage.^114^ To further reduce toxicity and improve systemic circulation, the MIPs can also be coated with a hydrophilic, biocompatible coating, such as polyethene glycol (PEG), which minimises opsonin adsorption, thereby extending their blood circulation, reducing their toxicity, and improving their performance.^26^

While these strategies improve the biological performance of MIPs, translating them from laboratory-scale synthesis to large-scale production introduces additional challenges related to reproducibility, yield, and regulatory compliance. Current synthesis procedures fail to reproducibly control polymerisation and lead to low yield, which does not facilitate large-scale, reproducible MIPs manufacturing within the complex regulatory health framework.^256^ There are a few MIPs reactor designs reported in literature, none of which are commercially available or widely implemented within the community, likely because they still suffer from low yield and cumbersome operation.^257,258^

Adaptation of platforms used for polymer synthesis in other research areas facilitates *in situ* monitoring of critical polymerisation parameters (*e.g.* size, molecular weight) and *in silico* and experimental screening of the polymer parameter space holds promise for automating and scaling up synthesis.^259,260^ However, this requires a significant shift to how MIPs are currently produced, especially for complex proteins that are targeted in cancer drug delivery, with a need towards automating the full process from rational design to optimise composition, to high-throughput production and subsequent screening of specificity and selectivity for the target to link structural properties and components to overall binding affinity.^261^

Route of administration is another underexplored but critical consideration. Intravenous delivery requires MIPs with excellent colloidal stability, controlled protein corona formation, and efficient clearance to minimise long-term accumulation. Oral delivery, by contrast, imposes additional constraints. Orally administered nanoMIPs must protect the drug payload from the acidic gastric environment and digestive enzymes, maintain structural stability across the gastrointestinal pH gradient, and avoid premature drug release before reaching the absorption site.^262^ Efficient transport across the intestinal epithelium is also required, potentially *via* uptake by M cells in Peyer's patches or enterocyte-mediated transcytosis.^263^ In addition, strategies to reduce hepatic first-pass metabolism, such as promoting lymphatic uptake or surface functionalisation that enhances epithelial transport, may further improve systemic bioavailability.^262,264^ However, the effectiveness of this approach may be more feasible for small-molecule or small-peptide therapeutics, as degradation by digestive enzymes in the gastrointestinal tract remains a significant barrier for protein-based drugs within the MIPs structure.^193^ These divergent requirements underscore the need for application-specific MIPs design principles rather than universal carrier architectures.

## 6. Summary and future perspective

MIPs have progressed from niche recognition materials to increasingly sophisticated nanoplatforms capable of selective targeting, controlled drug loading, and stimulus-responsive release.^26^ In the context of cancer drug delivery, MIPs offer a compelling combination of molecular selectivity, chemical robustness, and synthetic flexibility that distinguishes them from conventional polymeric carriers and biological target ligands. As highlighted throughout this review, advances in imprinting strategies, epitope selection, polymer chemistry, and hybrid material design have enabled MIPs to achieve antibody-like affinities for cancer-relevant receptors while retaining superior stability and lower production costs.

Recent studies demonstrate that MIPs can be engineered not only to recognise overexpressed cancer receptors and microenvironmental cues but also to actively participate in intracellular trafficking and controlled drug release.^8^ Epitope imprinting has emerged as a particularly powerful approach, enabling synthetic receptors to recapitulate biologically validated binding motifs while avoiding the instability and synthetic constraints associated with whole-protein templates. When combined with nanoscale architectures, such as core–shell structures, hollow capsules, or hybrid inorganic–organic composites, MIPs can integrate targeting, protection, and release functions within a single platform. These attributes position MIPs as adaptable carriers for precision drug delivery, including applications that require receptor-mediated uptake, intracellular delivery, or combinational therapeutic strategies.^265^

Experience with MIP-based sensing, assays, and separation technologies has established several design principles that are highly informative for therapeutic MIPs, though the transition is not a direct, one-to-one translation. In particular, diagnostic and extraction applications have highlighted the importance of minimising non-specific binding in complex media, maintaining binding-site accessibility through surface or epitope imprinting, and using rational monomer-template selection and controlled synthesis to improve selectivity and reproducibility. These lessons are directly relevant to therapeutic MIPs because non-specific adsorption in complex media can compromise receptor recognition, alter colloidal stability, and influence drug retention and release. Commercial solid-phase extraction products, such as SupelMIP^266^ cartridges, further demonstrate that selective and robust imprinting can be translated into real-world analytical workflows in complex matrices; however, these *in vitro* systems do not need to address the additional constraints that define therapeutic translation.^267^ In contrast to diagnostic MIPs, drug-delivery nanoMIPs must also satisfy stringent requirements relating to cytotoxicity, haemocompatibility, immune interactions, biodistribution, degradation, clearance, and reproducible *in vivo* performance. Accordingly, lessons from diagnostic and separation MIPs should be viewed as transferable design principles rather than as direct evidence of therapeutic readiness. Successful translation of therapeutic MIPs will therefore require that molecular recognition be integrated from the outset with antifouling surface design, pharmacokinetic control, and systematic *in vivo* validation.

Stimuli-responsive MIPs represent one of the most promising directions for future development, particularly systems responsive to endogenous tumour characteristics such as acidic pH, redox imbalance, or enzymatic activity.^206,218^ Even though these systems align well with the intrinsic heterogeneity of TME, the TME is highly complex and dynamic, characterised by abnormal vasculature, hypoxia, altered extracellular matrix composition, immune suppression, and dynamic biochemical concentrations.^268^ Designing MIPs architecture that remains effective across this heterogeneity, while maintaining predictable release kinetics and therapeutic windows, remains a major scientific and engineering challenge.^129,269^

One potential strategy to address this challenge would be the development of theranostics MIP-based nanoplatforms that integrate diagnostic and therapeutic functionalities within a single system. DDSs are increasingly engineered with imaging capabilities, such as fluorescent probes, magnetic resonance imaging (MRI) contrast agents, nuclear imaging tracers for positron emission tomography (PET) and single-photon emission computed tomography (SPECT) or surface-enhanced Raman scattering (SERS) reporters, to enable tracking of nanoparticle biodistribution and therapeutic response.^270–272^ Incorporating analogous sensing or imaging elements into MIPs could enable real-time analysis of local TME characteristics and modulate the drug release kinetics accordingly. Such approaches may enhance treatment robustness across heterogeneous tumours while supporting imaging-guided therapy and improved patient stratification.

The integration of MIPs into combinational therapeutic platforms further expands their potential impact. As reviewed here, MIPs have been successfully combined with photothermal, photodynamic, magnetic, and immunomodulatory modalities, enabling synergistic therapies that address drug resistance and enhance tumour eradication. These multifunctional systems illustrate how molecular imprinting can be leveraged not only for targeting and delivery but also for reprogramming tumour biology and the surrounding microenvironment. Nonetheless, increasing system complexity must be balanced against manufacturability and translational feasibility, particularly when multiple functional components are incorporated into a single carrier.

Looking ahead, progress in MIPs-based drug delivery will depend on three converging developments. First, advances in rational design—supported by computational modelling, high-throughput screening, and machine learning—are expected to accelerate the identification of optimal monomer compositions, imprinting strategies, and release mechanisms. Second, innovations in reactor engineering and automated synthesis will be essential to achieve reproducible, scalable manufacturing that meets regulatory standards. Third, systematic *in vivo* studies, including pharmacokinetics, biodistribution, immunogenicity, and long-term safety, are required to bridge the gap between promising *in vitro* performance and clinical translation.

In the near term, the most likely entry point for MIPs into healthcare remains the diagnostic space, where regulatory barriers are lower and performance requirements differ from those of therapeutics. Success in diagnostics will play a critical role in building confidence in the safety, reliability, and scalability of MIPs technologies. In the long term, the continued integration of materials science, chemical engineering, and biomedical insights has the potential to establish MIPs as a new class of synthetic, precision-engineered drug delivery systems capable of addressing the complexity and heterogeneity of cancer therapy.

## Author contributions

Shreya Tiwari: writing – original draft, data curation, formal analysis. Charles Luke Hutchinson: writing – original draft, data curation, formal analysis. Pankaj Singla: writing – original draft, data curation, formal analysis. Robert C. Rintoul: writing – review and editing, conceptualisation, funding. Timothy H. Witney: writing – review and editing, conceptualisation, funding. Nicholas W. Turner: writing – original draft, writing – review and editing. Marloes Peeters: conceptualisation, supervision, funding, writing – review and editing.

## Conflicts of interest

The authors declare there are no conflicts of interest associated with this work.

## Declaration on generative AI use

No AI tools were used in the preparation of this manuscript or the acquisition of data.

## Data Availability

No primary research results, software or code have been included, and no new data were generated or analysed as part of this review.
